# Ilegal pero vigente: la gestación por subrogación desde el prisma moral de mujeres urbanas chinas en Nanjing

**DOI:** 10.18294/sc.2026.5995

**Published:** 2026-01-13

**Authors:** Zhenkang Li

**Affiliations:** 1 Candidata a Doctora en Derecho Médico, School of Civil and Commercial Law, Southwest University of Political Science and Law, Chongqing, China. lizhenkang12345@163.com Southwest University of Political Science and Law School of Civil and Commercial Law Southwest University of Political Science and Law Chongqing China lizhenkang12345@163.com

**Keywords:** Gestación Subrogada, Técnicas Reproductivas, Tecnología de Reproducción Asistida, China

## Abstract

La forma en que las mujeres urbanas chinas comprenden y evalúan moralmente la gestación por subrogación continúa siendo un área insuficientemente explorada, a pesar de la persistencia de esta práctica de manera clandestina bajo una prohibición legal estricta en China. Desde la perspectiva de la sociología moral, este estudio cualitativo pone en primer plano estas voces desatendidas mediante entrevistas semiestructuradas a 24 mujeres de Nanjing, realizadas entre mayo y julio de 2025. Los resultados muestran una forma de “ambivalencia pragmática”: las participantes no sostienen posturas binarias de aprobación o rechazo, sino que construyen una posición matizada y dependiente del contexto. Esta posición se configura a partir de tres dimensiones claves: 1) las experiencias reproductivas encarnadas intensifican la sensibilidad ética; 2) la presión por la fertilidad y las necesidades prácticas reproductivas impulsan un apoyo; y 3) la prohibición legal y las normas morales dominantes refuerzan el escepticismo estructural. Sostenemos que las actitudes de las mujeres no constituyen elecciones abstractas, sino que se sitúan en la tensión entre autonomía corporal y obligación familiar.

## Introducción

**Figura 1:**
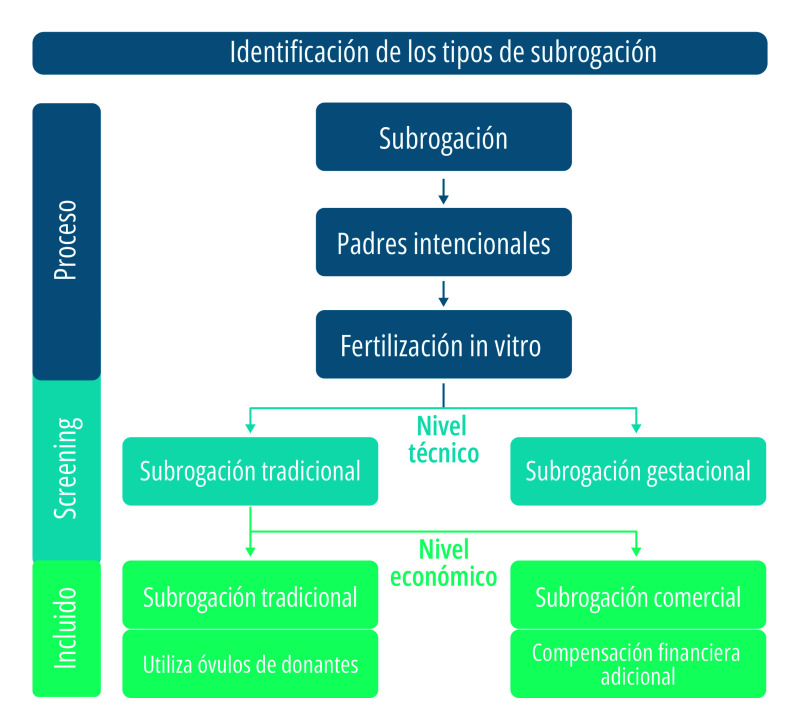
Proceso y clasificación de la subrogación.

En los últimos años, junto con el avance acelerado de las tecnologías de reproducción asistida (ART), la gestación por subrogación se ha instalado como un punto nodal de controversias éticas y debates académicos en China^1^. Se define como un acuerdo reproductivo en el que una mujer (gestante subrogante) lleva adelante un embarazo y da a luz a un niño o a una niña para otra persona o pareja, en función de un acuerdo previo. La subrogación suele clasificarse en dos modalidades: tradicional y gestacional (Figura 1). En la subrogación tradicional, la gestante aporta su propio material genético; en cambio, en la subrogación gestacional –hoy predominante en el mercado global– se emplea la fertilización in vitro (FIV) para implantar un embrión creado a partir de gametos de los padres intencionales o de donantes, lo que elimina el vínculo genético entre la gestante y el niño o la niña^2^.

Fuente: Elaboración propia.

Aunque la tecnología lo hace posible, la práctica se encuentra profundamente estratificada. En el plano económico, se distingue entre subrogación altruista (solo reembolso de gastos) y subrogación comercial (que incluye compensación financiera). Esta última concentra el mayor escrutinio moral, especialmente por las preocupaciones asociadas a la mercantilización del cuerpo de las mujeres y a la explotación del trabajo reproductivo^3^^,^^4^.

### El contexto chino: ilegal pero vigente

Existe una paradoja singular en torno a la práctica de la gestación por subrogación en China. A nivel macro, el gobierno sostiene una prohibición estricta de todas las formas de subrogación, al afirmar que esta práctica vulnera principios fundamentales del Código Civil de la República Popular China^5^. Sin embargo, a nivel micro, prospera un mercado comercial clandestino altamente lucrativo.

Estimaciones de la Comisión Nacional de Salud indican que la tasa de infertilidad en China se ubica entre el 7% y el 10%, lo que deja a más de 30 millones de personas enfrentando dificultades para concebir, de las cuales aproximadamente un 20% no podría lograr un embarazo sin la mediación de tecnologías de reproducción asistida^6^^,^^7^.

Impulsado por esta “ansiedad por la fertilidad” y por valores confucianos profundamente arraigados que subrayan la continuidad del linaje familiar, el mercado subterráneo de subrogación alcanzó una generación estimada de 2,31 mil millones de dólares estadounidenses en 2023, con proyecciones que ascenderían a 3,90 mil millones de dólares hacia 2030^8^. En el marco de los cambios demográficos en China y el descenso sostenido de la tasa de natalidad, la subrogación se ha instalado como un dilema urgente para la gobernanza y la ética social.

El discurso académico existente sobre la gestación por subrogación se ha concentrado, de manera predominante, en la gobernanza legal y en debates éticos a nivel macro^9^. La producción científica global suele clasificar los modelos de regulación en: prohibición integral, regulación altruista o legalización condicionada. En el contexto chino, la literatura ha discutido extensamente los argumentos a favor y en contra de la legalización, la regulación de los acuerdos transfronterizos y la determinación judicial del estatus parental^10^^,^^11^^,^^12^^,^^13^.

Sin embargo, persiste un vacío crítico en la investigación empírica acerca de cómo las mujeres urbanas chinas –como potenciales participantes u observadoras– comprenden y evalúan moralmente la subrogación.

Para abordar esta brecha, el presente estudio se inscribe en la tradición teórica de la sociología moral. A diferencia del abordaje ético abstracto, aquí conceptualizamos el “juicio moral” como una evaluación situada: un proceso en el que las personas negocian valores en tensión dentro de contextos sociales específicos. Examinar la subrogación desde la perspectiva de mujeres urbanas chinas es particularmente relevante, dado que los ideales confucianos sobre la maternidad, el linaje y el orden familiar las posicionan en el cruce entre la responsabilidad reproductiva y las expectativas familiares. Sus percepciones no solo expresan concepciones sobre el nacimiento, la maternidad y la autonomía corporal, sino que también revelan cómo las políticas estatales y las normas morales dominantes son reinterpretadas, discutidas y negociadas en la vida cotidiana^14^^,^^15^.

### La investigación actual: las voces de mujeres urbanas chinas en Nanjing

Nanjing constituye un sitio de investigación particularmente pertinente por diversas razones. En términos geográficos, se ubica en una zona de transición estratégica entre el norte y el sur de China, y ha funcionado históricamente como un corredor clave para la movilidad poblacional y el intercambio cultural, posición que ha favorecido la integración sostenida de tradiciones culturales septentrionales y meridionales^16^.

Al mismo tiempo, la ciudad conserva un fuerte arraigo en estructuras familiares tradicionales y en la ética confuciana de parentesco, situando a las mujeres en el centro de la tensión entre la autonomía reproductiva moderna y las obligaciones familiares tradicionales. A partir del análisis de las narrativas de mujeres urbanas de orígenes sociales y trayectorias diversas, este estudio busca ofrecer una perspectiva multidimensional sobre la subrogación y la protección de los derechos de las mujeres, así como aportar insumos empíricos a los debates teóricos y normativos sobre la gobernanza de la gestación por subrogación y la salvaguarda de los derechos reproductivos en países en desarrollo.

## Métodos

### Diseño del estudio

Situado en un paradigma constructivista, desde un abordaje cualitativo, se realizaron entrevistas semiestructuradas a 24 mujeres urbanas chinas con trayectorias y orígenes diversos en Nanjing. Este enfoque fue seleccionado para recuperar perspectivas auténticas y nociones morales situadas en torno a la gestación por subrogación.

### Procedimiento de reclutamiento y participantes

Antes de iniciar las entrevistas formales, se desarrolló una fase preparatoria de tres semanas. Esta etapa incluyó la planificación del diseño de investigación, la definición del muestreo y la puesta a prueba piloto de la guía de entrevista.

Posteriormente, se difundió un aviso de reclutamiento en la plataforma de redes sociales Xiaohong Shu (Little Red Book), mediante el cual se invitó públicamente a mujeres residentes en Nanjing a participar en el estudio. Para reflejar la realidad demográfica de la urbanización acelerada en China, la noción de “mujeres urbanas” adoptada aquí no se limita a residentes nativas. La muestra incluyó, de manera intencional, a mujeres provenientes de otras regiones que migraron a Nanjing por motivos educativos o laborales, en tanto se encuentran actualmente integradas al tejido social de la ciudad.

Con el propósito de abarcar un amplio rango de perspectivas, se utilizó un muestreo intencional siguiendo una estrategia de máxima variación. Las potenciales participantes fueron evaluadas para garantizar diversidad en edad (rango: 18 a 40 años), estado civil, y nivel socioeconómico. Finalmente, se incorporaron 24 mujeres al estudio. Las características sociodemográficas se presentan de manera agregada en la Tabla 1.

**Tabla 1:** Características sociodemográficas de las mujeres participantes. Ciudad de Nanjing, China (mayo–julio, 2025).

No.	Edad	Nivel educativo	Lugar de origen de la familia	Estado civil (casada)	Estado reproductivo (tiene hijas/os)
1	18	Universitario incompleto	Nanjing	No	No
2	22	Universitario incompleto	Anhui	No	No
3	24	Posgrado (maestría) incompleto	Shanghai	No	No
4	24	Posgrado (maestría) incompleto	Anhui	No	No
5	21	Tecnicatura superior completa	Anhui	Sí	No
6	29	Posgrado (maestría) completo	Nanjing	Sí	Sí
7	37	Universitario completo	Nanjing	Sí	Sí
8	26	Posgrado (maestría) completo	Shandong	No	No
9	27	Posgrado (maestría) completo	Jiangxi	Sí	Sí
10	30	Posgrado (maestría) completo	Shanxi	Sí	No
11	33	Tecnicatura superior completa	Jiangsu	Sí	Sí
12	18	Universitario incompleto	Jiangsu	No	No
13	20	Universitario incompleto	Nanjing	No	No
14	27	Tecnicatura superior completa	Heilongjiang	No	No
15	25	Posgrado (maestría) incompleto	Nanjing	Sí	Sí
16	40	Secundario completo	Jiangsu	Sí	Sí
17	36	Posgrado (maestría) completo	Anhui	Sí	Sí
18	37	Universitario completo	Anhui	No	No
19	26	Universitario completo	Fujian	Sí	No
20	28	Posgrado (maestría) completo	Henan	No	No
21	25	Posgrado (maestría) incompleto	Hubei	No	No
22	23	Posgrado (maestría) incompleto	Chongqing	Sí	No
23	19	Universitario incompleto	Beijing	No	No
24	23	Universitario completo	Nanjing	Sí	Sí

Fuente: Elaboración propia.

### Entrevistas

Los datos se recolectaron mediante entrevistas individuales presenciales, cara a cara, realizadas en idioma chino entre mayo y julio de 2025 en Nanjing. Cada entrevista tuvo una duración aproximada de 45 a 60 minutos. A partir de una guía semiestructurada, se invitó a las participantes a compartir sus fuentes de información, sus actitudes y sus concepciones en torno a la gestación por subrogación. Las preguntas centrales se diseñaron con un orden progresivo desde percepciones generales hacia evaluaciones éticas específicas, incluyendo interrogantes como: “¿Cómo percibís la actual prohibición legal de la subrogación?” y “¿Qué conflictos éticos considerás que emergen en la subrogación comercial?”. Todas las entrevistas fueron grabadas en audio con autorización previa, y posteriormente transcritas de manera literal, palabra por palabra (verbatim).

### Análisis de los datos

El análisis siguió el proceso de seis fases propuesto por Braun y Clarke^17^ para el análisis temático. El proceso inició con la familiarización, mediante lecturas reiteradas de las transcripciones hasta alcanzar una inmersión profunda en el corpus. Luego, se realizó codificación minuciosa línea por línea, destacando rasgos semánticos relevantes (por ejemplo: “miedo al dolor”, “ansiedad por el linaje”). Los códigos se agruparon en subtemas, por ejemplo, los códigos referidos a riesgos físicos fueron reunidos bajo la categoría “vulnerabilidad corporal”. En la fase de revisión temática, se construyó un árbol de codificación (*coding tree*) para visualizar relaciones entre códigos y subtemas. Finalmente, se definieron tres ejes temáticos principales (Tabla 2) capaces de expresar la “ambivalencia pragmática” de las participantes, y se integraron citas textuales ilustrativas para sustentar la síntesis interpretativa. Para fortalecer la confiabilidad de los datos, se realizó la devolución a las participantes (*member checking*) una vez finalizadas las entrevistas, permitiendo –a quienes así lo desearan– revisar y validar las principales interpretaciones de sus relatos.

**Tabla 2:** Proceso de codificación: de los datos crudos a los temas. Ciudad de Nanjing, China (mayo–julio, 2025).

Citas ilustrativas	Códigos iniciales	Subtemas	Temas principales
“*Dar a luz es mucho más doloroso de lo que la gente imagina… Realmente no puedo imaginar atravesar tanto sufrimiento y luego entregar al bebé*”. (P11)	a) Intensidad del dolor de parto. b) Límites físicos del cuerpo	El trauma del nacimiento	Tema 1: Las experiencias reproductivas encarnadas intensifican la sensibilidad ética
“*Pensar en una gestante subrogante haciendo todo esto para otra persona me genera incomodidad… La subrogación convierte ese sufrimiento en una transacción*”. (P17)	a) Empatía con la gestante subrogante. b) Rechazo del sufrimiento transaccional	Empatía encarnada	(Igual al anterior)
“*Si una pareja realmente no puede concebir, la subrogación puede ser su única esperanza… es una responsabilidad*”. (P9)	a) Último recurso ante la infertilidad. b) Obligación moral de tener hijos/as	Linaje y responsabilidad	Tema 2: La presión por la fertilidad y las necesidades prácticas reproductivas impulsan un apoyo condicional
“*La gente a mi alrededor dice que la subrogación es inmoral… Bajo esa presión moral es muy difícil apoyarla abiertamente*”. (P16)	a) Miedo al juicio social. b) Conformidad con las normas sociales	Estigma social	Tema 3: La prohibición legal y las normas morales dominantes refuerzan el escepticismo estructural
“*El gobierno lo prohíbe claramente… automáticamente pensás que debe haber serios problemas éticos detrás*”. (P12)	a) La ley como guía moral. b) Confianza en la regulación estatal	Internalización de la ley	(Igual al anterior)

Fuente: Elaboración propia.

### Reflexividad y posicionamiento

Dado que la investigación involucra experiencias corporales sensibles, reconocemos la co-construcción del sentido en el proceso analítico. La entrevistadora principal es una investigadora mujer con formación en Derecho Médico, posicionamiento que favoreció la construcción de confianza y la apertura dialógica en temas íntimos de la reproducción. Para contrarrestar posibles sesgos derivados de conocimientos legales previos, se sostuvo un registro reflexivo sistemático mediante un diario de campo y se adoptó una posición de “neutralidad empática”, priorizando la lógica experiencial de las participantes por sobre lecturas críticas de tipo jurídico-normativo.

### Aspectos éticos

Este estudio fue evaluado y aprobado por el Comité de Ética del Centro de Investigación en Derecho Médico (N.º de aprobación MLRC-2025-021) y se ajustó a los principios de la Declaración de Helsinki. Todas las participantes fueron informadas de manera completa sobre los objetivos, los procedimientos y la confidencialidad del estudio, y aceptaron participar de manera voluntaria. Se obtuvo consentimiento informado por escrito de todas las participantes antes de la recolección de los datos. En los procesos de registro y análisis de los relatos, se aplicaron protocolos rigurosos de anonimización: no se conservaron datos identificatorios.

## Resultados

El análisis muestra que las actitudes de mujeres urbanas chinas frente a la gestación por subrogación no constituyen posiciones binarias de apoyo u oposición simple. En su lugar, las participantes elaboran una forma compleja de “ambivalencia pragmática”, en la que el razonamiento moral depende de los contextos y de las situaciones. Esta ambivalencia se construye en la intersección dinámica de tres dimensiones claves: 1) las experiencias reproductivas encarnadas intensifican la sensibilidad ética; 2) la presión por la fertilidad y las necesidades prácticas reproductivas impulsan un apoyo; y 3) la prohibición legal y las normas morales dominantes refuerzan el escepticismo estructural.

### Las experiencias reproductivas encarnadas intensifican la sensibilidad ética

Un tema recurrente en las entrevistas fue la profunda influencia de la experiencia corporal –en particular, el embarazo, el parto y la recuperación posparto– en la construcción de juicios morales. A diferencia del razonamiento moral abstracto, las participantes que habían atravesado un parto activaron formas de empatía encarnada, interpretando el trabajo reproductivo de la gestante subrogante desde su propia vulnerabilidad física.

Para muchas, la intensidad extrema del dolor de parto operó como un umbral que dificulta aceptar la subrogación como una transacción. La participante 11, que ya había sido madre, expresó la imposibilidad de concebir ese sufrimiento como algo mercantilizable:

“*Dar a luz es mucho más doloroso de lo que la gente imagina… En la sala de partos sentís que llegás a tu límite. Realmente no puedo imaginar atravesar tanto sufrimiento y luego entregar al bebé. Sentiría el corazón roto por la madre subrogante*”. (P11)

Más allá del dolor, las participantes señalaron los riesgos médicos sostenidos y la desigualdad moral implicada en transferir esa carga a otra mujer. Esto sugiere que la resistencia no nace de una posición abstracta, sino de un reconocimiento compartido de vulnerabilidad femenina. La participante 17, por ejemplo, subrayó que la comercialización del parto le resulta intrínsecamente injusta:

“*Después de dar a luz, mi primer pensamiento fue que no todas pueden soportar esto… La subrogación convierte este sufrimiento en una transacción, y eso me resulta injusto para las mujeres*”. (P17)

En la misma línea, la participante 7 afirmó que la comprensión plena del riesgo –que solo emerge tras la experiencia– vuelve inquietante la idea de subrogación:

“*Recién después de dar a luz entendí cuánto sufre una mujer… Pensar en una subrogante haciendo todo esto para otra persona me genera incomodidad. Por eso no apoyo la subrogación*”. (P7)

### La presión por la fertilidad y las necesidades prácticas reproductivas impulsan un apoyo condicional

Si bien las experiencias corporales alimentaron posiciones de resistencia, los imperativos sociales habilitaron un relato de apoyo condicionado. Esta aceptación no se funda en una aprobación ideológica de la subrogación, sino en el reconocimiento pragmático de la “ansiedad por la fertilidad” y del mandato cultural de continuidad del linaje. En este marco, la subrogación es resignificada como un “mal necesario” o como una solución pragmática frente al sufrimiento social.

Diversas participantes subrayaron que, en el contexto chino, tener hijos/as suele interpretarse como una obligación familiar antes que como una elección personal. Esta presión cultural abre un espacio de empatía hacia parejas que enfrentan la infertilidad. Como lo expresó la participante 9:

“*En muchas familias, especialmente en las tradicionales, tener un/a hijo/a no es solo algo personal: es una responsabilidad. Si una pareja realmente no puede concebir, la subrogación podría ser su única esperanza… Puedo comprender por qué la considerarían*”. (P9)

Esta posición pragmática también se ve reforzada por el escrutinio social que recae sobre mujeres sin hijos/as. La subrogación aparece así como un mecanismo para aliviar presiones comunitarias. La participante 15 señaló:

“*En nuestra cultura, si no tenés hijos/as, los familiares y vecinos/as preguntan todo el tiempo, y la presión se vuelve enorme… Para parejas con dificultades de infertilidad, la subrogación podría ser una forma de aliviar esa carga*”. (P15)

La necesidad médica (por ejemplo, riesgos de salud que impiden el embarazo) otorga, además, una legitimidad específica a la subrogación, al diferenciarla de subrogaciones motivadas por razones de “vanidad” (sin indicación médica). La participante 6 argumentó:

“*Si una mujer tiene problemas de salud y un embarazo sería peligroso, la subrogación se convierte en una solución práctica… Es preferible a poner en riesgo su propia vida*”. (P6)

### La prohibición legal y las normas morales dominantes refuerzan el escepticismo estructural

El tercer eje temático muestra cómo la prohibición legal estatal opera como una brújula moral para la ciudadanía. Las participantes tienden a interiorizar la prohibición como indicio de una inmoralidad intrínseca de la subrogación, evidenciando el modo en que la gobernanza modula éticas privadas. Incluso quienes expresan empatía frente a la infertilidad regresan a una posición escéptica por la “señal moral” que emite la ley. La participante 13 describió la ley como un límite que estructura la percepción moral:

“*Como es ilegal en China, la mayoría de las personas ya lo ve como algo que no debería hacerse. Aunque alguien pueda comprender las razones… la ley te hace sentir que es moralmente incorrecto o riesgoso*”. (P13)

Este escepticismo legal se ve reforzado por normas culturales tradicionales que privilegian la maternidad “natural”. La creencia de que la identidad materna se forja a través de la gestación continúa siendo dominante. La participante 3 subrayó el estigma social que recae sobre maternidades no gestacionales:

“*Nuestra sociedad cree que una madre debe gestar a su propio/a hijo/a. Si no lo hace, dirán que es antinatural o irresponsable… incluso cuando intento tener una mente abierta, igual me genera incomodidad*”. (P3)

Además, la convergencia entre la prohibición legal estatal y la moral social dominante genera una “espiral de silencio”, en la que expresar apoyo público a la subrogación se vuelve socialmente riesgoso. La participante 6 observó:

“*La gente a mi alrededor dice que la subrogación es inmoral… Bajo esa presión moral, es muy difícil apoyarla abiertamente. Te preocupa que piensen mal de vos*”. (P6)

## Discusión

Este estudio muestra que las actitudes de las mujeres frente a la gestación por subrogación en Nanjing no constituyen juicios fijos, sino negociaciones dinámicas configuradas por la interacción entre experiencias reproductivas encarnadas, presiones por la fertilidad y normas legales-morales. Conceptualizamos esta complejidad actitudinal como “ambivalencia pragmática”, un marco conceptual que explica cómo las mujeres transitan la tensión entre la aversión ética a la mercantilización y la necesidad social de continuidad del linaje^18^^,^^19^.

### Relacionalidad encarnada y similitudes estructurales

Un hallazgo central es la gravitación de la experiencia corporal en la elaboración de juicios éticos. Las participantes jerarquizan la realidad visceral de la vulnerabilidad física e interpretan la subrogación desde un reconocimiento de “sufrimiento compartido”, antes que desde abstracciones normativas sobre derechos^20^^,^^21^. Esto dialoga con la noción de “reproducción estratificada” documentada en contextos latinoamericanos, donde el trabajo reproductivo de mujeres en posiciones sociales desventajadas es problematizado de manera análoga desde preocupaciones por la integridad corporal y la explotación^22^^,^^23^.

A diferencia de los discursos del feminismo liberal que ponen el acento en el “contrato” y la “elección”, nuestros resultados indican que las mujeres urbanas chinas –especialmente aquellas que atravesaron un parto– objetan la mercantilización del útero porque comprenden de manera íntima los costos físicos implicados en esa transacción^24^. Esto sugiere que la ética reproductiva en China, al igual que en otros países en desarrollo, se funda en una relacionalidad encarnada. El trauma del parto opera como un puente empático con la gestante subrogante, y tensiona la idea de que la lógica de mercado pueda eludir la materialidad biológica de la reproducción^25^^,^^26^.

### Ambivalencia pragmática: entre la autonomía y el linaje

Introducimos el término “ambivalencia pragmática” para describir la tensión estructural entre el malestar ético individual y la racionalidad social colectiva. Las participantes manifestaron una dualidad matizada: por un lado, expresaron una preocupación profunda por la integridad corporal de la gestante subrogante; por otro, reconocieron el peso abrumador de las obligaciones familiares^27^.

En una sociedad donde “sostener la continuidad del linaje familiar” continúa siendo un mandato cultural potente, la subrogación es resignificada: deja de aparecer exclusivamente como un “mal comercial” para ser entendida como un “último recurso pragmático”. Esta negociación refleja hallazgos en otros contextos del Sur Global, donde las mujeres frecuentemente se ven compelidas a relativizar la defensa de su autonomía corporal para responder a expectativas reproductivas asociadas a roles de género tradicionales^28^^,^^29^.

El análisis muestra que los juicios morales no son absolutos, sino que se producen dentro de una trama de relaciones familiares y presiones económicas. El “apoyo condicionado” observado no es adhesión plena, sino un compromiso: una negociación entre la mujer moderna que reivindica derechos y la hija o esposa que enfrenta mandatos reproductivos anclados en expectativas tradicionales^30^.

### El Estado como arquitecto moral: una perspectiva biopolítica

El estudio también clarifica el rol del Estado en la construcción activa de moralidades privadas. En China, la prohibición legal estricta de la subrogación opera como una “señal moral proactiva”, que encuadra la práctica como moralmente ilegítima antes de cualquier deliberación individual^31^. Esto es consistente con una lectura biopolítica de la gobernanza reproductiva, donde el Estado no solo regula volúmenes poblacionales, sino la legitimidad de los métodos reproductivos.

Las participantes a menudo interiorizaron la prohibición como evidencia de ilegitimidad moral, lo que permite observar cómo la gobernanza estatal tamiza y modula razonamientos éticos individuales^32^. Asimismo, los ideales confucianos de orden familiar refuerzan esta cautela y contribuyen a la percepción de que la subrogación pone en riesgo relaciones de parentesco. En este sentido, las actitudes de las mujeres chinas no constituyen meras preferencias personales, sino que se producen en la intersección entre biopolítica estatal, tradicionalismo cultural y la experiencia universal de vulnerabilidad corporal^33^.

## Conclusión

Este estudio indica que las actitudes de las mujeres urbanas chinas frente a la gestación por subrogación expresan una forma de “ambivalencia pragmática”. Lejos de constituir un conjunto fragmentado de puntos de vista individuales, esta actitud surge de la compleja interacción entre experiencias corporales, presiones reproductivas de carácter social y normas legales y éticas prevalentes. En términos de formulación de políticas, resulta imperativo trascender la lógica de una prohibición generalizada de un enfoque único y uniforme del tipo “*one-size-fits-all*”. En su lugar, la gobernanza debería atender los dilemas que enfrentan las mujeres entre las cargas reproductivas y la vulnerabilidad física. Este enfoque encarna una filosofía centrada en las personas, que respeta los derechos reproductivos de las mujeres y expresa un cuidado humanista, garantizando así que las políticas sean legalmente compatibles y, al mismo tiempo, estén ancladas en las realidades sociales.

Estos hallazgos ofrecen aportes significativos para el Sur Global: al enfrentar conflictos entre la ética reproductiva y el derecho, resulta crucial equilibrar el bienestar de las mujeres, la responsabilidad social y los marcos legales, evitando modelos de gobernanza rígidos que desatienden las necesidades individuales. Como señaló el presidente Xi, “la aspiración del pueblo a una vida mejor es aquello por lo que luchamos”^34^. En consecuencia, las políticas reproductivas deben centrarse en la protección de la salud y la dignidad de las mujeres, haciendo que la gobernanza sea más humana y efectiva.
