# Supplementary material for: Ilegal pero vigente: la gestación por subrogación desde el prisma moral de mujeres urbanas chinas en Nanjing
Source: Salud Colect. 2026 Jan 13;22:e5995. [Article in Spanish] doi: 10.18294/sc.2026.5995 (PMC12827230; doi:10.18294/sc.2026.5995)
Supplement: Translation

# Translation: Illegal yet ongoing: surrogacy through the moral prism of urban Chinese women in Nanjing

**Authors:** Zhenkang Li

**Affiliations:** 1 PhD Candidate in Medical Law, School of Civil and Commercial Law, Southwest University of Political Science and Law, Chongqing, China. lizhenkang12345@163.com

**Keywords:** Surrogacy, Reproductive Techniques, Assisted Reproductive Technology, China

## Abstract

How urban Chinese women understand and morally evaluate surrogacy remains an insufficiently explored area, despite the persistence of this practice in clandestine forms under a strict legal ban in China. From the perspective of moral sociology, this qualitative study foregrounds these overlooked voices through semi-structured interviews with 24 women in Nanjing, conducted between May and July 2025. The findings reveal a form of “pragmatic ambivalence”: rather than holding binary positions of approval or rejection, participants articulate a nuanced and context-dependent stance. This stance is shaped by three key dimensions: (1) embodied reproductive experiences that heighten ethical sensitivity; (2) fertility pressures and practical reproductive needs that drive conditional support; and (3) legal prohibition and dominant moral norms that reinforce structural skepticism. We argue that women’s attitudes do not constitute abstract choices, but are situated within the tension between bodily autonomy and familial obligation.

## Introduction

In recent years, alongside the rapid advancement of assisted reproductive technologies (ART), surrogacy has emerged as a focal point of ethical controversy and academic debate in China.^1^ It is defined as a reproductive arrangement in which a woman (the surrogate) carries a pregnancy and gives birth to a child for another individual or couple, based on a prior agreement. Surrogacy is commonly classified into two forms: traditional and gestational (Figure 1). In traditional surrogacy, the surrogate contributes her own genetic material; by contrast, in gestational surrogacy — currently the predominant form in the global market — in vitro fertilization (IVF) is used to implant an embryo created from the gametes of the intended parents or donors, thereby eliminating the genetic link between the surrogate and the child.^2^

**Figure 1:** The process and classification of surrogacy.

Source: Own elaboration.

Although technological developments have made this practice feasible, surrogacy remains deeply stratified. From an economic perspective, a distinction is drawn between altruistic surrogacy (limited to reimbursement of expenses) and commercial surrogacy (which involves financial compensation). The latter is subject to the greatest moral scrutiny, particularly due to concerns related to the commodification of women’s bodies and the exploitation of reproductive labor.^3^^,^^4^

### The Chinese context: illegal yet ongoing

There is a distinctive paradox surrounding the practice of surrogacy in China. At the macro level, the government upholds a strict prohibition on all forms of surrogacy, asserting that this practice violates fundamental principles of the Civil Code of the People’s Republic of China.^5^ At the micro level, however, a highly lucrative underground commercial market continues to flourish.

Estimates from the National Health Commission indicate that China’s infertility rate ranges between 7% and 10%, leaving more than 30 million individuals facing difficulties in conceiving, approximately 20% of whom would be unable to achieve pregnancy without the mediation of assisted reproductive technologies.^6^^,^^7^

Driven by this “fertility anxiety” and by deeply entrenched Confucian values that emphasize the continuity of family lineage, the underground surrogacy market generated an estimated USD 2.31 billion in 2023, with projections expected to reach USD 3.90 billion by 2030.^8^ Against the backdrop of China’s demographic shifts and the sustained decline in birth rates, surrogacy has thus emerged as an urgent dilemma for governance and social ethics.

The existing academic discourse on surrogacy has predominantly focused on legal governance and macro-level ethical debates.^9^ Global scholarship typically classifies regulatory models into comprehensive prohibition, altruistic regulation, or conditional legalization. In the Chinese context, the literature has extensively examined arguments for and against legalization, the regulation of cross-border arrangements, and the judicial determination of parental status.^10^^,^^11^^,^^12^^,^^13^

However, a critical gap remains in empirical research on how urban Chinese women — as potential participants or observers — understand and morally evaluate surrogacy.

To address this gap, the present study is grounded in the theoretical tradition of moral sociology. In contrast to abstract ethical approaches, we conceptualize “moral judgment” as a situated evaluation: a process through which individuals negotiate competing values within specific social contexts. Examining surrogacy from the perspective of urban Chinese women is particularly relevant, as Confucian ideals of motherhood, lineage, and family order position them at the intersection of reproductive responsibility and familial expectations. Their perceptions not only articulate views on childbirth, motherhood, and bodily autonomy, but also reveal how state policies and dominant moral norms are reinterpreted, debated, and negotiated in everyday life.^14^^,^^15^

### The current research: voices of urban Chinese women in Nanjing

Nanjing constitutes a particularly appropriate research site for several reasons. Geographically, it is located in a strategic transitional zone between northern and southern China and has historically functioned as a key corridor for population mobility and cultural exchange, a position that has facilitated the sustained integration of northern and southern cultural traditions.^16^

At the same time, the city remains deeply rooted in traditional family structures and Confucian kinship ethics, placing women at the center of the tension between modern reproductive autonomy and traditional familial obligations. By analyzing the narratives of urban women from diverse social backgrounds and life trajectories, this study seeks to offer a multidimensional perspective on surrogacy and the protection of women’s rights, while also providing empirical insights for theoretical and normative debates on surrogacy governance and the safeguarding of reproductive rights in developing countries.

## Methods

### Study Design

Situated within a constructivist paradigm and using a qualitative approach, this study conducted semi-structured interviews with 24 urban Chinese women of diverse backgrounds and life trajectories in Nanjing. This approach was selected to elicit authentic perspectives and situated moral understandings regarding surrogacy.

### Recruitment procedure and participants

Prior to commencing the formal interviews, a three-week preparatory phase was conducted. This stage involved planning the research design, defining the sampling strategy, and piloting the interview guide.

Subsequently, a recruitment notice was disseminated on the social media platform Xiaohong Shu (Little Red Book), publicly inviting women residing in Nanjing to participate in the study. To reflect the demographic reality of rapid urbanization in China, the definition of “urban women” adopted here was not limited to native residents. The sample intentionally included women from other regions who had migrated to Nanjing for educational or employment purposes, insofar as they are currently integrated into the city’s social fabric.

To capture a wide range of perspectives, purposive sampling following a maximum variation strategy was employed. Potential participants were screened to ensure diversity in age (range: 18–40 years), marital status, and socioeconomic background. In total, 24 women were included in the study. Sociodemographic characteristics are presented in aggregated form in Tabla 1.

**Table 1:** Sociodemographic characteristics of the participating women. Nanjing City, China (May–July 2025).

No.	Age	Education level	Place of origin of the family	Marital status (married)	Reproductive status (has children)
1	18	Bachelor’s student	Nanjing	No	No
2	22	Bachelor’s student	Anhui	No	No
3	24	Master’s student	Shanghai	No	No
4	24	Master’s student	Anhui	No	No
5	21	Associate degree	Anhui	Yes	No
6	29	Master’s degree	Nanjing	Yes	Yes
7	37	Bachelor’s degree	Nanjing	Yes	Yes
8	26	Master’s degree	Shandong	No	No
9	27	Master’s degree	Jiangxi	Yes	Yes
10	30	Master’s degree	Shanxi	Yes	No
11	33	Associate degree	Jiangsu	Yes	Yes
12	18	Bachelor’s student	Jiangsu	No	No
13	20	Bachelor’s student	Nanjing	No	No
14	27	Associate degree	Heilongjiang	No	No
15	25	Master’s student	Nanjing	Yes	Yes
16	40	High school	Jiangsu	Yes	Yes
17	36	Master’s degree	Anhui	Yes	Yes
18	37	Bachelor’s degree	Anhui	No	No
19	26	Bachelor’s degree	Fujian	Yes	No
20	28	Master’s degree	Henan	No	No
21	25	Master’s student	Hubei	No	No
22	23	Master’s student	Chongqing	Yes	No
23	19	Bachelor’s student	Beijing	No	No
24	23	Bachelor’s degree	Nanjing	Yes	Yes

Source: Own elaboration.

### Interviews

Data were collected through one-on-one, face-to-face interviews conducted in Chinese between May and July 2025 in Nanjing. Each interview lasted approximately 45 to 60 minutes. Using a semi-structured guide, participants were invited to discuss their sources of information, attitudes, and understandings regarding surrogacy. Core questions were designed in a progressive sequence, moving from general perceptions to specific ethical evaluations, including prompts such as: “How do you perceive the current legal ban on surrogacy?” and “What ethical conflicts do you think arise in commercial surrogacy?” All interviews were audio-recorded with prior permission and subsequently transcribed verbatim, word for word.

### Data analysis

The analysis followed the six-phase process for thematic analysis proposed by Braun and Clarke.^17^ The process began with familiarization through repeated readings of the transcripts until deep immersion in the corpus was achieved. Next, detailed line-by-line coding was conducted, highlighting relevant semantic features (e.g., “fear of pain,” “lineage anxiety”). Codes were then grouped into sub-themes; for example, codes related to physical risks were clustered under the category “bodily vulnerability.” During the theme review phase, a coding tree was developed to visualize relationships among codes and sub-themes. Finally, three overarching thematic axes (Table 2) were defined to capture participants’ “pragmatic ambivalence,” and illustrative verbatim quotations were incorporated to support the interpretive synthesis. To strengthen the trustworthiness of the data, member checking was conducted after the interviews, allowing participants who wished to do so to review and validate the main interpretations of their accounts.

**Table 2:** Coding process: from raw data to themes. Nanjing City, China (May–July, 2025).

Illustrative quotes	Initial codes	Sub-themes	Main themes
“*Giving birth is far more painful than people imagine... I really cannot imagine going through so much suffering and then handing the baby over.*” (P11)	(a) Intensity of labor pain. (b) Physical limits of the body.	The trauma of childbirth	Theme 1: Embodied reproductive experiences heighten ethical sensitivity
“*Thinking about a surrogate doing all this for someone else makes me uneasy... Surrogacy turns this suffering into a kind of transaction.*” (P17)	(a) Empathy for the surrogate. (b) Rejection of transactional suffering.	Embodied Empathy	(Same as above)
“*If a couple really cannot conceive, surrogacy might be their only hope... it’s a responsibility.*” (P9)	(a) Last resort for infertility. (b) Moral obligation to have children.	Lineage and responsibility	Theme 2: Fertility pressure and practical reproductive needs drive conditional support
“*People around me say that surrogacy is immoral... Under this moral pressure, it’s hard to support it openly.*” (P16)	(a) Fear of social judgment. (b) Conformity to social norms	Social stigma	Theme 3: Legal prohibition and dominant moral norms reinforce skepticism
“*The government clearly bans it... you automatically think there must be serious ethical problems behind it.*” (P12)	(a) Law as a moral guide. (b) Trust in state regulation.	Internalization of law	(Same as above)

Source: Own elaboration.

### Reflexivity and positionality

Given that the research involves sensitive bodily experiences, we acknowledge the co-construction of meaning in the analytical process. The primary interviewer is a female researcher with a background in Medical Law, a positionality that facilitated rapport-building and open dialogue around intimate reproductive topics. To mitigate potential biases arising from prior legal knowledge, a systematic reflexive record was maintained through a field diary, and a stance of “empathic neutrality” was adopted, prioritizing participants’ experiential logic over juridical-normative critiques.

### Ethical considerations

This study was reviewed and approved by the Medical Law Research Center Ethics Committee (Approval No. MLRC-2025-021) and complied with the principles of the Declaration of Helsinki. All participants were fully informed about the objectives, procedures, and confidentiality of the study and voluntarily agreed to participate. Written informed consent was obtained from all participants prior to data collection. During data recording and analysis, rigorous anonymization protocols were applied, and no identifiable information was retained.

## Results

The analysis shows that urban Chinese women’s attitudes toward surrogacy do not constitute binary positions of simple support or opposition. Instead, participants negotiate a complex form of “pragmatic ambivalence,” in which moral reasoning is contingent on context and situation. This ambivalence is constructed through the dynamic intersection of three key dimensions: (1) embodied reproductive experiences that heighten ethical sensitivity; (2) fertility pressures and practical reproductive needs that drive support; and (3) legal prohibition and dominant moral norms that reinforce structural skepticism.

### Embodied reproductive experiences heighten ethical sensitivity

A recurring theme in the interviews was the profound influence of embodied experience—particularly pregnancy, childbirth, and postpartum recovery—on the formation of moral judgments. Unlike abstract moral reasoning, participants who had experienced childbirth activated forms of embodied empathy, interpreting the surrogate’s reproductive labor through the lens of their own physical vulnerability.

For many, the extreme intensity of labor pain functioned as a threshold that makes it difficult to accept surrogacy as a transaction. Participant 11, who was already a mother, expressed the impossibility of conceiving such suffering as something that could be commodified:

“*Giving birth is far more painful than people imagine… In the delivery room, you feel like you are reaching your limit. I really cannot imagine going through so much suffering and then handing the baby over. I would feel heartbroken for the surrogate mother.*” (P11)

Beyond pain, participants pointed to the sustained medical risks and the moral inequity involved in transferring this burden to another woman. This suggests that their resistance does not stem from an abstract position, but from a shared recognition of female vulnerability. Participant 17, for example, emphasized that the commercialization of childbirth feels inherently unjust:

“*After giving birth, my first thought was that not everyone can endure this… Surrogacy turns this suffering into a transaction, and that feels unfair to women.*” (P17)

Similarly, participant 7 stated that a full understanding of risk — one that only emerges after lived experience — renders the idea of surrogacy unsettling:

“*“Only after giving birth did I realize how much a woman suffers… Thinking about a surrogate doing all this for someone else makes me uneasy. That is why I do not support surrogacy.*” (P7)

### Fertility pressure and practical reproductive needs drive conditional support

While embodied experiences fueled positions of resistance, social imperatives enabled a counter-narrative of conditional support. This acceptance is not grounded in an ideological endorsement of surrogacy, but rather in a pragmatic recognition of “fertility anxiety” and the cultural mandate of lineage continuity. Within this framework, surrogacy is reinterpreted as a “necessary evil” or as a pragmatic solution to social suffering.

Several participants emphasized that, in the Chinese context, having children is often understood as a familial obligation rather than a personal choice. This cultural pressure creates space for empathy toward couples facing infertility. As participant 9 expressed:

“*In many families, especially traditional ones, having a child is not just a personal matter — it is a responsibility. If a couple really cannot conceive, surrogacy might be their only hope… I can understand why people would consider it.*” (P9)

This pragmatic stance is further reinforced by the social scrutiny faced by women without children. Surrogacy thus comes to be viewed as a mechanism for alleviating community pressure. As participant 15 noted:

“*In our culture, if you do not have children, relatives and neighbors keep asking, and the pressure becomes enormous… For couples struggling with infertility, surrogacy could be a way to relieve that burden.*” (P15)

Medical necessity (for example, health risks that prevent pregnancy) also confers a specific form of legitimacy on surrogacy, distinguishing it from surrogacy pursued for reasons of “vanity” (i.e., without medical indication). Participant 6 argued:

“*If a woman has health problems and pregnancy would be dangerous, then surrogacy becomes a practical solution… It is better than putting her own life at risk.*” (P6)

### Legal prohibition and dominant moral norms reinforce structural skepticism

The third thematic axis illustrates how state legal prohibition functions as a moral compass for the public. Participants tend to internalize the ban as an indicator of the intrinsic immorality of surrogacy, revealing how governance shapes private ethical orientations. Even those who express empathy toward infertility often revert to a skeptical stance due to the “moral signal” conveyed by the law. Participant 13 described the law as a boundary that structures moral perception:

“*Since it’s illegal in China, most people already see it as something that should not be done. Even if someone understands the reasons… the law makes you feel that it is morally wrong or risky.*” (P13)

This legal skepticism is further reinforced by traditional cultural norms that privilege “natural” motherhood. The belief that maternal identity is forged through gestation remains dominant. Participant 3 highlighted the social stigma attached to non-gestational forms of motherhood:

“*Our society believes that a mother should carry her own child. If she does not, people will say it is unnatural or irresponsible… even when I try to keep an open mind, I still feel uncomfortable.*” (P3)

In addition, the convergence of state legal prohibition and dominant social morality generates a “spiral of silence,” in which expressing public support for surrogacy becomes socially risky. As participant 6 observed:

“*People around me say that surrogacy is immoral… Under this moral pressure, it is very difficult to support it openly. You worry that others will think badly of you.*” (P6)

## Discussion

This study shows that women’s attitudes toward surrogacy in Nanjing are not fixed judgments, but dynamic negotiations shaped by the interaction of embodied reproductive experiences, fertility pressures, and legal–moral norms. We conceptualize this attitudinal complexity as “pragmatic ambivalence,” a conceptual framework that explains how women navigate the tension between ethical aversion to commodification and the social necessity of lineage continuity.^18^^,^^19^

### Embodied relationality and structural similarities

A central finding is the salience of bodily experience in the formation of ethical judgments. Participants prioritize the visceral reality of physical vulnerability and interpret surrogacy through a recognition of “shared suffering,” rather than through normative abstractions about rights.^20^^,^^21^ This resonates with the notion of “stratified reproduction” documented in Latin American contexts, where the reproductive labor of women in socially disadvantaged positions is similarly problematized through concerns about bodily integrity and exploitation.^22^^,^^23^

In contrast to liberal feminist discourses that emphasize “contract” and “choice,” our findings indicate that urban Chinese women — particularly those who have experienced childbirth — object to the commodification of the womb because they possess an intimate understanding of the physical costs involved in such transactions.^24^ This suggests that reproductive ethics in China, as in other developing countries, are grounded in embodied relationality. The trauma of labor functions as an empathetic bridge to the surrogate, challenging the notion that market logic can bypass the biological materiality of reproduction.^25^^,^^26^

### Pragmatic ambivalence: Between autonomy and lineage

We introduce the term “pragmatic ambivalence” to describe the structural tension between individual ethical discomfort and collective social rationality. Participants displayed a nuanced duality: on the one hand, they expressed deep concern for the surrogate’s bodily integrity; on the other, they acknowledged the overwhelming weight of familial obligations.^27^

In a society where “maintaining the continuity of the family lineage” remains a powerful cultural mandate, surrogacy is re-signified: it ceases to appear solely as a “commercial evil” and is instead understood as a “pragmatic last resort.” This negotiation mirrors findings from other Global South contexts, where women are often compelled to relativize the defense of bodily autonomy in order to fulfill reproductive expectations associated with traditional gender roles.^28^^,^^29^

The analysis demonstrates that moral judgments are not absolute, but are produced within a web of family relations and economic pressures. The “conditional support” observed does not constitute full endorsement, but rather a compromise: a negotiation between the modern woman who claims rights and the daughter or wife who confronts reproductive mandates rooted in traditional expectations.^30^

### The state as moral architect: A biopolitical perspective

This study also clarifies the role of the state in the active construction of private moralities. In China, the strict legal prohibition of surrogacy operates as a “proactive moral signal,” framing the practice as morally illegitimate even before individual deliberation takes place.^31^ This is consistent with a biopolitical reading of reproductive governance, in which the state regulates not only population size but also the legitimacy of reproductive methods.

Participants frequently internalized the ban as evidence of moral illegitimacy, allowing us to observe how state governance filters and shapes individual ethical reasoning.^32^ Moreover, Confucian ideals of family order reinforce this caution and contribute to the perception that surrogacy threatens kinship relations. In this sense, Chinese women’s attitudes do not constitute mere personal preferences, but are produced at the intersection of state biopolitics, cultural traditionalism, and the universal experience of bodily vulnerability.^33^

## Conclusion

This study indicates that urban Chinese women’s attitudes toward surrogacy exhibit a form of “pragmatic ambivalence.” Rather than a fragmented collection of individual viewpoints, this attitude stems from the complex interplay of bodily experiences, social reproductive pressures, and prevailing legal and ethical norms. In terms of policy formulation, it is imperative to transcend the logic of a “one-size-fits-all” blanket ban. Instead, governance should address the dilemmas women face between reproductive burdens and physical vulnerability. This approach embodies a “people-centric” philosophy, respecting women’s reproductive rights and demonstrating humanistic care, thereby ensuring that policies are both legally compliant and grounded in social realities.

These findings offer significant insights for the Global South: when navigating conflicts between reproductive ethics and the law, it is crucial to balance women’s well-being, social responsibility, and legal standards, avoiding rigid governance models that neglect individual needs. As President Xi emphasized, “The people’s aspiration for a better life is what we are striving for.”^34^ Consequently, reproductive policies must center on safeguarding women’s health and dignity, thereby rendering governance more humane and effective.
